# Dupilumab improve acquired reactive perforating collagenosis characterized by type 2 inflammation

**DOI:** 10.3389/fimmu.2023.1240262

**Published:** 2023-08-10

**Authors:** Ben Liu, Yibei Wu, Xiaoyan Wu, Xinyu Zhong, Ruzeng Xue, Zhenying Zhang

**Affiliations:** ^1^ Department of Dermatology, The Eighth Affliated hospital of Sun Yat-sen University, Shenzhen, Guangdong, China; ^2^ Department of Dermatology, Dermatology Hospital, Southern Medical University, Guangzhou, China

**Keywords:** ARPC, dupilumab, Th2, IL4, IL13

## Abstract

**Background:**

Acquired reactive perforating collagenosis (ARPC) is a clinically challenging disease with an unclear pathogenesis.

**Objective:**

To evaluate the efficacy and safety of dupilumab for the treatment of ARPC, and analyze the expression of type 2 inflammation-related molecules in ARPC lesions.

**Methods:**

This retrospective cohort study included 20 patients with ARPC; 10 received dupilumab and 10 received conventional therapy. The efficacy and safety of dupilumab were evaluated at 12 weeks. Immunohistochemical and immunofluorescence analyses of T- and B-cell markers, and type 2 inflammation-related cytokines, were performed on skin samples from ARPC patients, atopic dermatitis (AD) patients, and healthy controls.

**Results:**

Significantly more patients showed improvements in the Investigator Global Assessment score (100% vs. 0%; p < 0.0001) and itching (90%/8.33%, P =.001) in the dupilumab group compared to the conventional group at 12 weeks. There were no adverse effects in the dupilumab group. The ARPC lesions showed enhanced dermal infiltration of CD3+ T-cells, with a predominance of Th2 cells, similar to AD lesions. IL-4 and IL-13 were co-localized with GATA3 in ARPC lesions.

**Conclusion:**

Dupilumab improved ARPC charaterized with type 2 inflammation.

## Introduction

Acquired reactive perforating collagenosis (ARPC) is a type of perforating dermatosis characterized by transepidermal penetration and the elimination of necrotic collagen. It presents with multiple, widespread, skin-colored or erythematous dome-shaped umbilicated papules and nodules of variable sizes with a scab-like central plug on the trunk, limbs, and face, accompanied by intense pruritus. In some patients, the Koebner phenomenon is present (1). The typical histopathological manifestation of ARPC is a dome-shaped lesion with epidermal ulceration and a central crater that extends from the epidermis to the reticular dermis. These lesions contain keratin, poly-morphonuclear cell debris, and altered collagen in vertical strands, and are also characterized by an accumulation of neutrophils, lymphocytes, histiocytes, and cellular debris (2).

The pathogenesis of ARPC is unclear but is likely multifactorial. ARPC was previously thought to be associated with microangiopathy and impaired elimination of metabolic products because it typically affects patients with diabetes or chronic kidney disease (3). Hypoxia and diabetes mellitus can cause collagen detachment and interkeratinocyte disruption (2, 4). Mehregan et al. (5, 6) suggested that trauma in genetically susceptible individuals leads to collagen necrobiosis in the dermal papillae. Moreover, immune activation may be involved in the development of ARPC. Collagen types IV and VII from the basal membrane in the plug of ARPC skin lesions (7) induce an abnormal immune response. Transforming growth factor (TGF)-β, an important pleiotropic immune regulator, is overexpressed in ARPC lesions (8, 9). TGF-β in gingival fibroblasts is upregulated by interleukin (IL)-13, which is a T-helper type 2 (Th2) cytokine (10). However, it is unclear whether type 2 inflammation is involved in the pathogenesis of ARPC.

The management of ARPC is challenging. Topical keratolytic agents, narrowband ultraviolet B therapy, antihistamines, and topical and systemic corticosteroids (11) have been used to treat ARPC; however, no treatment has been approved. Dupilumab, which is a fully human monoclonal antibody against interleukin‐4 receptor α (IL‐4Rα), effectively treats pruritic disorders. Our research group first reported the successful use of Dupilumab for the treatment of ARPC last year (12), subsequently, scholars from South Korea, Saudi Arabia, and Spain have also demonstrated therapeutic effects of dupilumab for ARPC (13–15), suggesting the involvement of Th2 inflammation in the pathophysiology of the disease.

We performed a retrospective cohort study of ARPC patients to evaluate the efficacy and safety of dupilumab monotherapy and conventional therapy. In addition, we explored the infiltration of T and B cells, as well as the expression of Th2-related cytokines in ARPC lesions, to characterize the inflammation associated with the disease.

## Materials and methods

### Study design and participants

In this retrospective cohort study, data of ARPC patients were collected from The Eighth Affiliated Hospital of Sun Yat-sen University and Dermatology Hospital of Southern Medical University between December 2020 and December 2022. The study enrolled 10 ARPC patients who received dupilumab and 10 age-, sex-, and disease severity-matched ARPC patients who received conventional drugs. The ARPC patients were aged > 18 years and had umbilicated papules or nodules with a central adherent keratotic plug, as well as histopathological findings of elimination of necrotic basophilic collagen tissue into a cup‐shaped epidermal depression (3). We reviewed the medical records and photographs of patients, and to extracted data on demographic factors, comorbidities, lesion characteristics, histological and immunological findings, laboratory test results, treatments, responses to the initial treatment at 4 and 12 weeks, and adverse events (AEs) during the 12-week follow up. Missing data were obtained from patients by telephone. The study protocol was approved by the Ethics Committee and Institutional Review Board of the participating study centers. Written informed consent was obtained from the study participants.

### Efficacy and safety of dupilumab for ARPC

The primary outcome was the W12 Investigator Global Assessment (IGA) score (0 or 1), and the secondary outcomes were the W4 IGA score (0 or 1), W4 numerical rating scale (NRS) score for itch (reduction ≥ 4 grades), and W12 NRS score for itch (reduction ≥ 4 grades). IGA 0 for ARCP was defined as the absence of umbilicated papules or nodules, while IGA1 was defined as having no more than 5 umbilicated papules or nodules in this study refering to the criteria for Investigator’s Global Assessment of Chronic Prurigo (16).The IGA rating was assessed by two dermatologists who are blinded to the patient’s enrollment status, basing on clinical photographs.

### Immunohistochemistry

Immunohistochemistry was performed to detect lymphocyte subsets and Th2-related molecules in skin samples of 22 patients with ARPC, 5 patients with atopic dermatitis (AD), and 5 healthy controls (HCs).

Formalin-fixed, paraffin-embedded 3-µm tissue sections were prepared for immunohistochemical analysis. The sections were incubated with the following primary antibodies: anti-CD3 (DF6594; Affinity Biosciences, Cincinnati, OH, USA), anti-CD20 (DF13319; Affinity Biosciences), anti-T-bet (ab150440; Abcam, Cambridge, UK), anti-GATA3 (ab199428; Abcam), anti-IL-4 (66142-1-Ig; Proteintech, Chicago, IL, USA), and anti-IL-13 (CSB-PA011590LA01HU; Cusabio, Wuhan, China). A mouse and rabbit antibody kit (KIT9922; MXB, Fuzhou, China) was used for the detection of primary antibodies.

Stained sections were scanned using the Pannoramic Scan 150 device (3DHISTECH Ltd., Budapest, Hungary), to produce digital images (40× and 200× magnification) that were imported into the CaseViewer 2.4 (3DHISTECH Ltd.) image management system. ImageJ software (NIH, Bethesda, MD, USA) was used to analyze the images. The levels of IL-4, IL-13, and T- and B-cell markers in the dermis are expressed in terms of cellular density.

### Immunofluorescence

Immunofluorescence staining for the Th2-related markers GATA3, IL-4, and IL-13 was performed on skin samples from five ARPC patients, three AD patients, and two HCs.

The skin sections were deparaffinized on a hot plate at 65°C for 1 h and then blocked with donkey serum (10%; SL050-10; Solarbio, Beijing, China) for 30 min at room temperature. Double staining was performed with GATA3 (ab199428; Abcam), IL4 (66142-1-Ig; Proteintech), GATA3 (ab199428; Abcam), and IL13 (CSB-PA011590LA01HU; Cusabio). After incubation with the primary antibodies, skin biopsy specimens were incubated with the secondary antibodies Alexa Fluor™ 488 and 594 (Invitrogen, Carlsbad, CA, USA). The nuclei were counterstained with DAPI. The stained sections were scanned using the Pannoramic Scan 150 device (3DHISTECH Ltd.), to obtain digital images that were imported into the CaseViewer 2.4 (3DHISTECH Ltd.) image management system.

### Statistical analysis

Normally distributed data are presented as means ± standard deviations (SDs). Student’s t-test and Fisher’s exact test were used to compare baseline variables between the groups. Primary and secondary outcomes were analyzed using Fisher’s exact test. Changes in IGA and NRS scores were analyzed using two-way repeated measures analysis of variance (ANOVA) and Tukey’s multiple comparison test. Immunohistochemical parameters are presented as means ± SDs, and were analyzed using one-way ANOVA and Tukey’s multiple comparison test. Student’s t-test, one-way ANOVA, and two-way repeated-measures ANOVA were performed using GraphPad Prism software (version 8.0.1; GraphPad Software Inc., San Diego, CA, USA). Fisher’s exact test was performed using SPSS software (version 25.0; IBM Corp., Armonk, NY, USA). P < 0.05 was considered indicative of statistical significance.

## Results

### Baseline characteristics

We divided 20 ARPC patients into dupilumab and conventional treatment groups. In the conventional treatment group, the patients received antihistamines and topical steroids; some patients also received thalidomide (n = 3) and narrowband ultraviolet B therapy (n = 1). Dupilumab was administered at an initial dose of 600 mg, followed by 2–12 doses of 300 mg every 2–4 weeks until symptomatic improvement was achieved. **Table 1** presents the baseline characteristics of the study participants. Age, gender, and baseline disease severity did not show statistically significant differences between two groups. Regarding comorbidities, there were 6 cases combined with diabetes, 2 cases with renal disease, 2 cases with AD, and 2 cases with allergic rhinitis (AR) in the Dupilumab group. While, in the traditional treatment group, only 1 case was combined with diabetes. All 10 patients in dupilumab group had previously received and failed in responsing to traditional treatments, such as topical or oral medication, phototherapy with narrowband ultraviolet B.

**Table 1:** Baseline characteristics of the patients in both groups.

Characteristic	Dupilumab group(n=10)	Conventional group(n=10)	P value
Mean age (SD), y	48.7(16.49)	59(17.98)	0.1985
Male/female,n	7/3	3/7	0.179
Comorbidities, n (%)			
Diabetes	6(60)	1(10)	0.057
Renal disease	2(20)	0	0.474
Atopic Dermatitis*	2(20)	0	0.474
Allergic Rhinitis	2(20)	0	0.474
Mean IGA(SD)	3.1 (0.88)	3.4 (0.7)	0.4083
Mean NRS(SD)	7.6 (1.43)	6.5 (1.08)	0.0681

*Two patients in this study were diagnosed as elderly atopic dermatitis, according to the criteria proposed by Williamson S et al. (17).

### Dupilumab is safe and effective for controlling itching and reducing lesion size in ARPC

**Figure 1:**
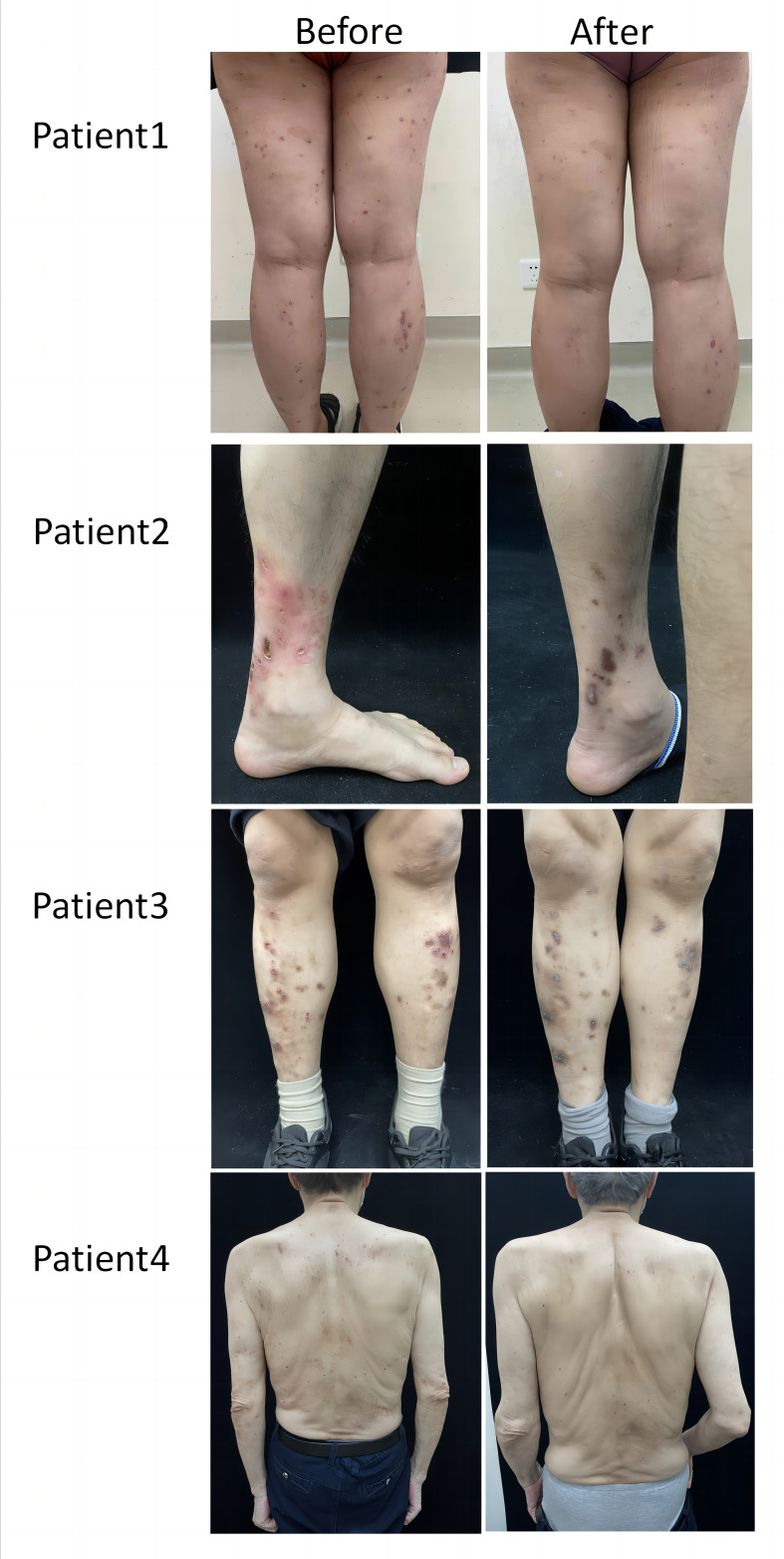
Representative clinical images of dupilumab group.

**Figure 2:**
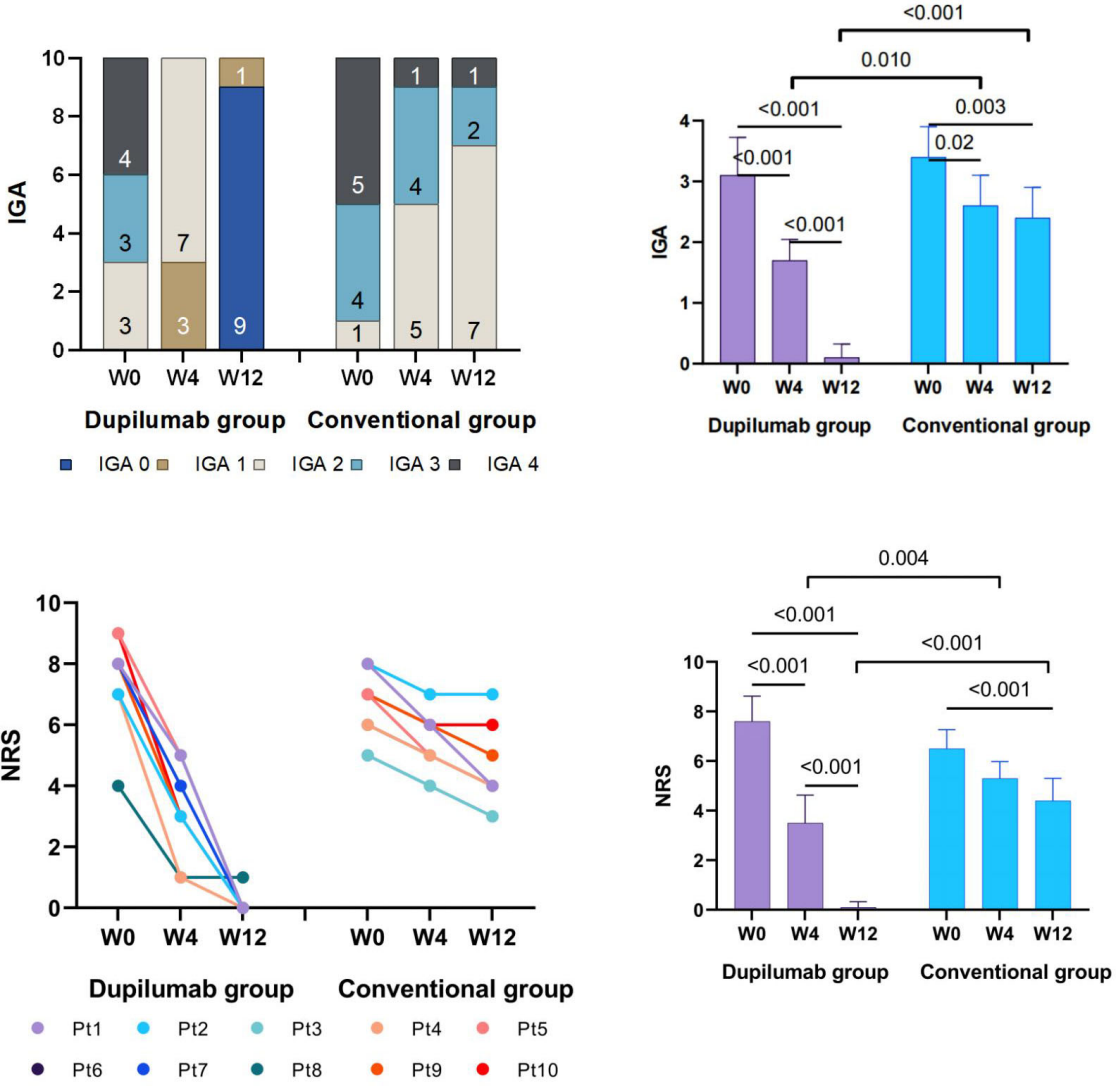
IGA and NRS change from baseline.

In the dupilumab group, patients experienced satisfactory clinical improvement after treatment (**Table 2**; **Figure 1**). At week 4, 3 (30%) of the 10 patients achieved an IGA score of 0 or 1, and 6 (60%) achieved a significant reduction (≥ 4 grades) in the NRS score for itch (mean decrease of 4.1 [from 7.6 to 3.5]). At week 12, all ten patients had achieved complete clearance of their skin lesions, and nine of ten (90%) achieved relief from pruritus, only one patient had slight itching, with a NRS score of 1. In the conventional group, no patient achieved an IGA score 0 or 1 at week 4 or 12 (**Figure 2**). Only 1 (10%) of the 10 patients in the conventional group achieved a significant reduction in the NRS score at 12 weeks; the mean NRS scores at baseline, week 4, and week 12 were 6.5, 5.3, and 4.4, respectively (**Figure 2**). We also conducted a statistical analysis excluding 4 patients with allergic diseases (2 cases with AD and 2 cases with AR), and the results are shown in **Table 3**. The most common AE was lethargy.

**Table 2:** Summary of efficacy outcomes.

Endpoint	Dupilumab group(n=10)	Conventional group(n=10)	P value
Patients achieving IGA0/1, %			
W4	30	0	0.049
W12	100	0	<0.001
Patients achieving NRS4, %			
W4	60	0	0.014
W12	90	10	0.001

NRS4, ≥4-point reduction in itch numerical rating scale score versus baseline; NRS4, ≥4-point reduction in itch numerical rating scale score versus baseline.

**Table 3:** Summary of efficacy outcomes, excluding patients with allergic diseases.

Endpoint	Dupilumab group(n=6)	Conventional group(n=10)	P value
Patients achieving IGA0/1, %			
W4	33.33	0	0.125
W12	100	0	<0.001
Patients achieving NRS4, %			
W4	50	0	0.036
W12	83.33	10	0.008

NRS4, ≥4-point reduction in itch numerical rating scale score versus baseline; NRS4, ≥4-point reduction in itch numerical rating scale score versus baseline.

AEs were more common in the conventional group (3/10) than dupilumab group (no AEs) during the 12 weeks of treatment. In the conventional group, two patients experienced lethargy and one experienced limb numbness.

### Overexpression of Th2-related molecules in ARPC lesions

**Figure 3:**
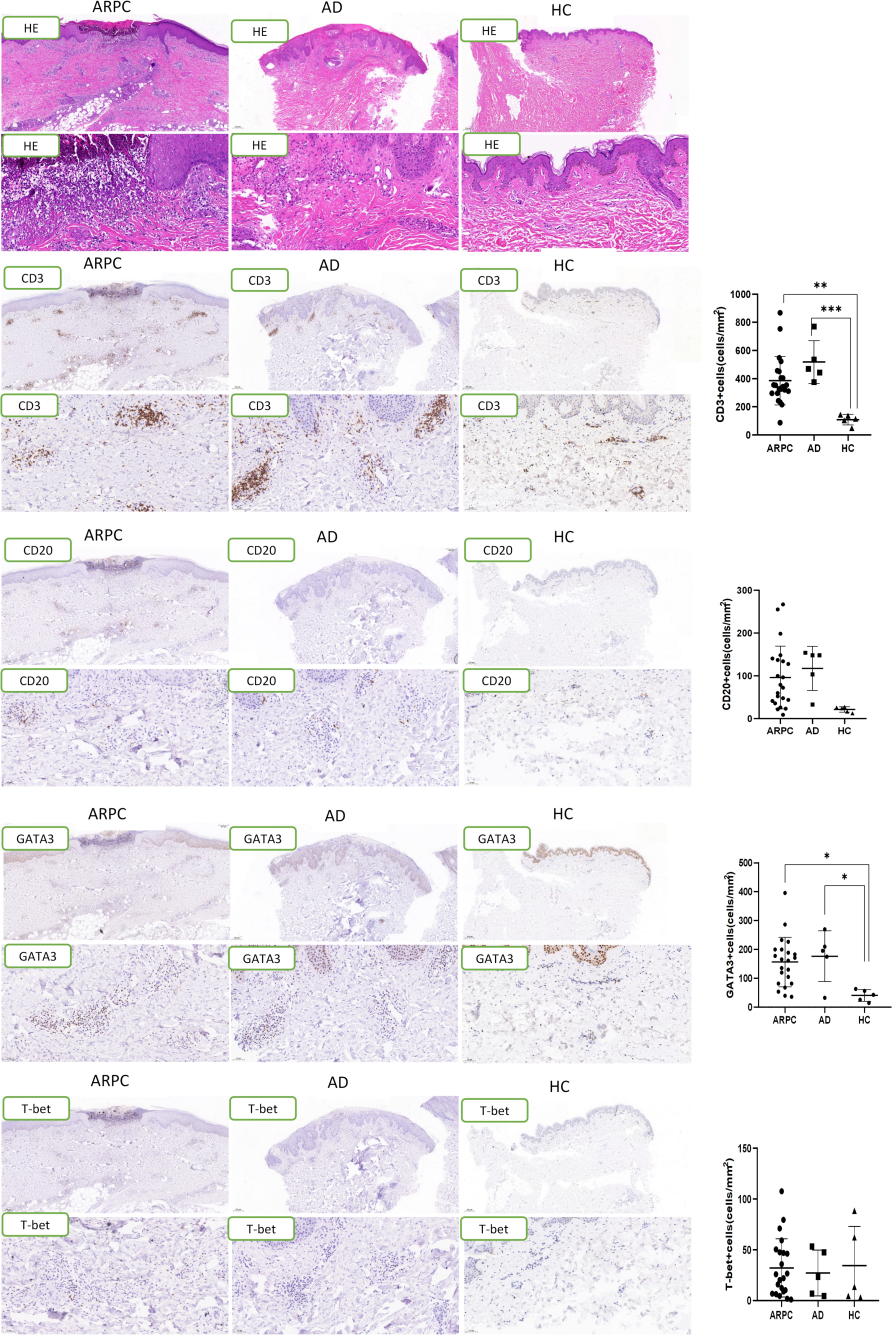
Representative images and data reporting hematoxylin and eosin staining, CD3, CD20, GATA3 and T-bet staining of APRC, AD and HC samples. Magnification: ×40, ×200, AD, atopic dermatitis, ARPC, acquired reactive perforating collagenosis, HC, healthy control, HE, hematoxylin and eosin. *P<0.05; **P<0.01; ***P<0.001.

Immunohistochemical staining was performed to analyze lymphocyte subsets in skin samples from ARPC patients, AD patients, and HCs. In AD samples, CD3+ cells were localized mainly in the superficial and mid dermis, whereas in ARPC samples CD3+ cells had infiltrated the entire dermis and subcutaneous tissue. The T-cell density in the dermis of ARPC was similar to that in AD and significantly higher than that in HCs (p = 0.0039 and = 0.0009, respectively; **Figure 3**). There were no significant differences among the groups in terms of CD20+ cell density. The skin samples were stained with T-bet and GATA3, which are the transcription factors of Th1 and Th2 cells, respectively. The GATA3+ cell density in the ARPC group was similar to that in the AD group and significantly higher than that in HCs (p = 0.0183 and = 0.0316, respectively). There were no significant differences among the groups in terms of T-bet+ cell infiltration. These results suggest a predominance of Th2 cells over Th1 cells in ARPC, similar to AD.

**Figure 4:**
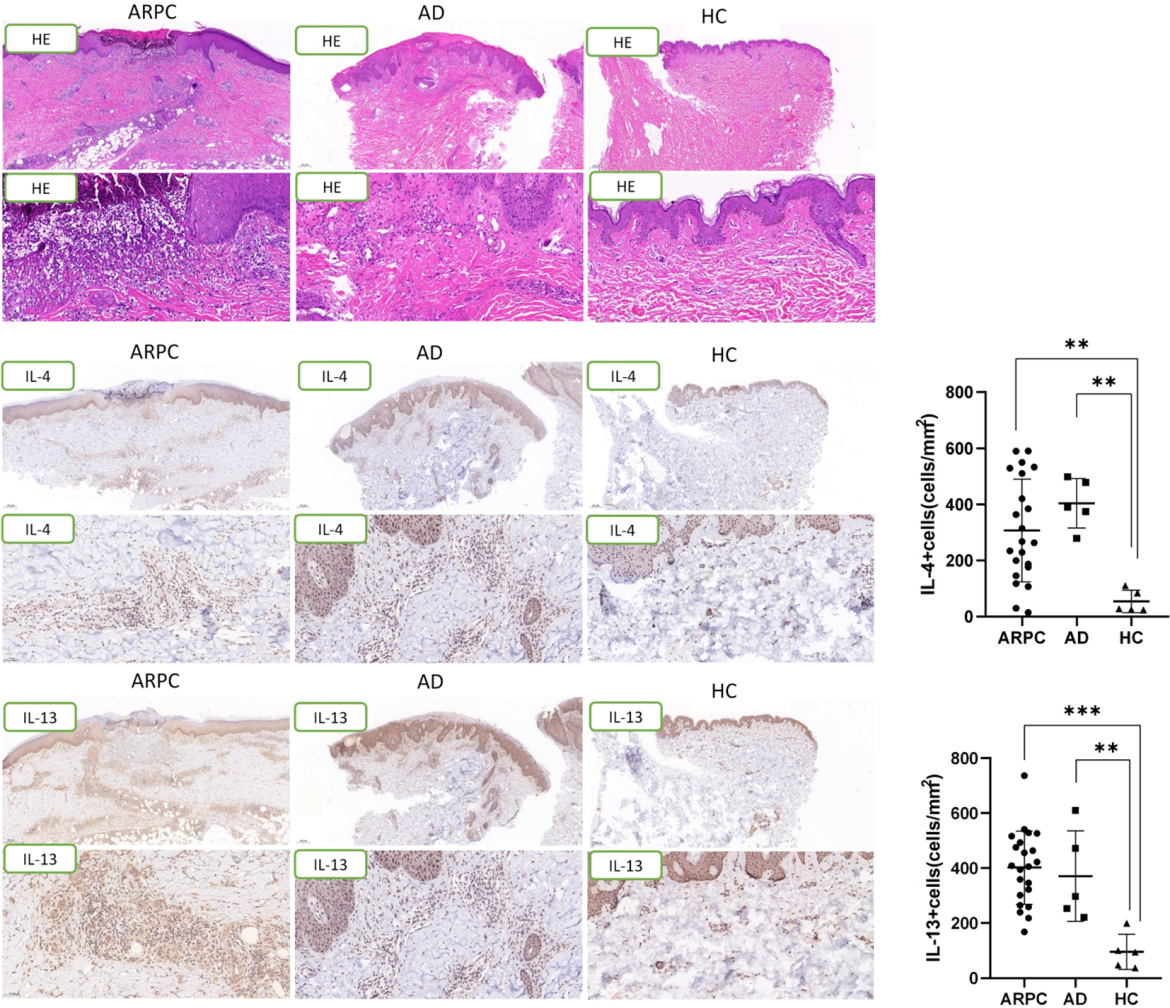
Representative images and data reporting hematoxylin and eosin staining, IL-4 and IL-13 staining of APRC, AD and HC samples. Magnification: ×40, ×200, AD, atopic dermatitis, ARPC, acquired reactive perforating collagenosis, HC, healthy control, HE, hematoxylin and eosin, IL, interleukin. **P<0.01; ***P<0.001.

**Figure 5:**
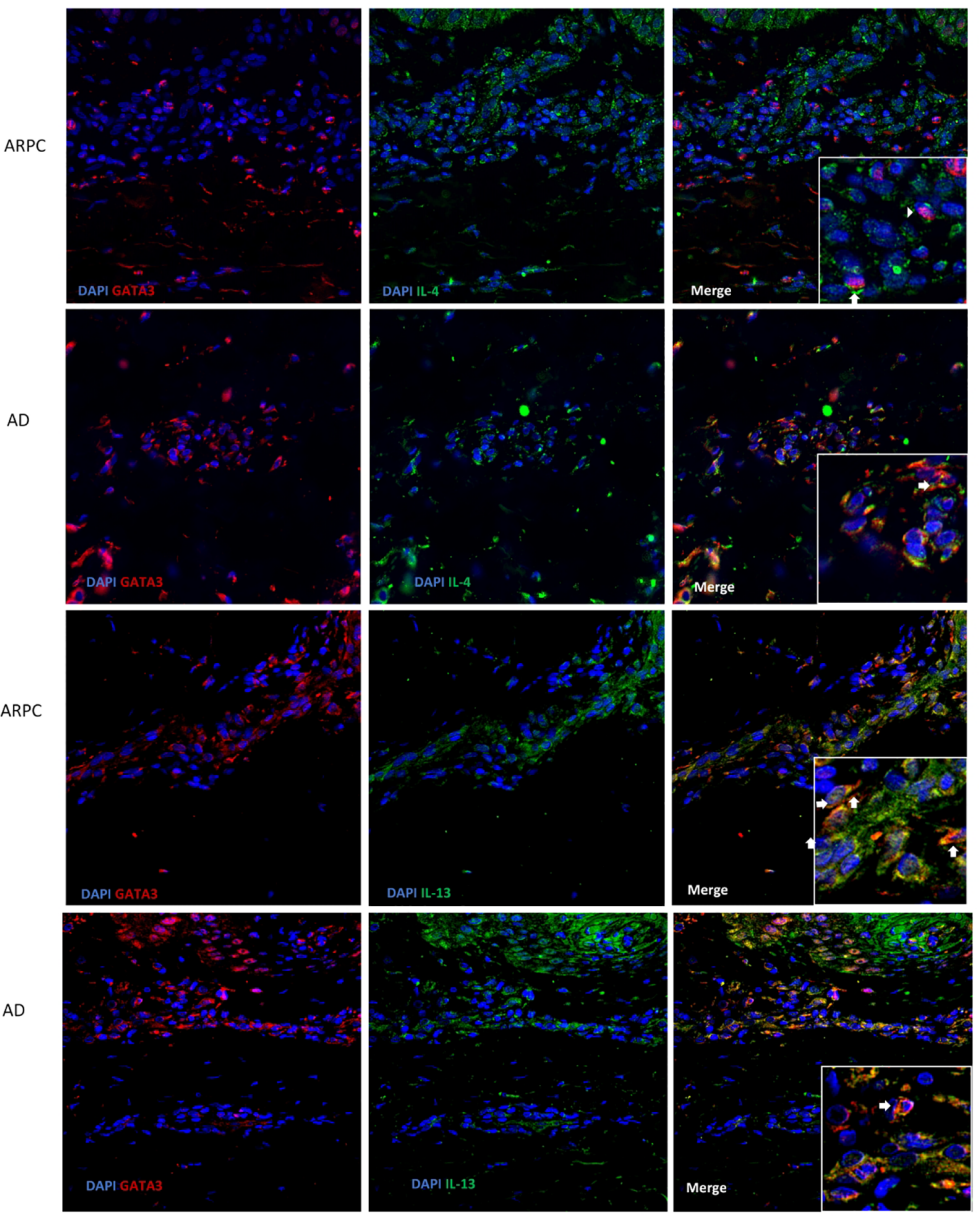
Representative images of a triple immunofluorescence staining of GATA3, IL-4 and IL-13 of ARPC and AD. Magnification: ×40, ×200. AD, atopic dermatitis, ARPC, acquired reactive perforating collagenosis, IL, interleukin.

The expression of Th2-associated cytokines, including IL-4 and IL-13, was analyzed by immunohistochemical staining of the ARPC, AD, and HC skin samples. The IL-4+ cell density was significantly higher in the ARPC and AD groups than in HCs (p = 0.0093 and = 0.0047, respectively) (**Figure 4**). Similarly, IL-13 overexpression was also observed in the ARPC and AD groups compared to HCs (p = 0.0002 and = 0.0066, respectively). Multi-immunofluorescence staining analysis suggested that IL-4 and IL-13 were co-expressed with the Th2 transcription factor GATA3 in ARCP and AD samples, suggesting that IL-4 and IL-13 were derived from GATA3+ Th2 cells in ARCP, similar to AD (**Figure 5**).

## Discussion

Dupilumab is a human monoclonal antibody against IL-4Ra, which regulates type 2 inflammation by inhibiting the IL-4 and IL-13 signaling pathways. Dupilumab improves itching in patients with prurigo nodularis, lichen planus, other pruritic disorders, and AD (18, 19). In the present retrospective cohort study, we evaluated the efficacy and safety of dupilumab for the treatment of ARPC. Dupilumab was significantly more effective than conventional treatment for ARPC. Dupilumab reduced the lesion size and improved pruritus within 4 weeks of treatment. After 12 weeks of treatment, the patients achieved clearance of skin lesions and relief from pruritus, with no AEs. Eight of the ten patients have been followed up for 2–12 months after dupilumab withdrawal and no cases of recurrence have been observed. Dupilumab was well-tolerated and effective in our patients, suggesting that type 2 inflammation might be involved in the pathogenesis of ARPC.

Previous studies of the pathogenesis of ARPC have focused on chronic venous insufficiency (20), systemic diseases (1), collagen degeneration (6), increased glycosylation products in skin lesions (21), and TGF-β3 overexpression during extracellular matrix remodeling and wound repair (9, 22). Few studies have evaluated the immune-related pathogenesis of ARPC. In the present study, we observed enhanced dermal infiltration of CD3+ T-cells with a predominance of Th2 cells in ARPC, similar to AD. Additionally, the cytokines IL-4 and IL-13, which act on neurons to promote itching, were also significantly upregulated in ARPC (18, 23). These findings suggest that Th2-type inflammation is involved in the pathogenesis of ARPC. We speculate that collagen degeneration induced by scratching or microangiopathy impaired the elimination of metabolic products, thereby triggering type 2 inflammation in the skin and promoting the development of ARPC.

In conclusion, Dupilumab is effective for ARPC and should be considered in cases of conventional treatment-resistant ARPC. Type 2 inflammation is involved in the pathogenesis of ARPC. Limited by the small sample size and absence of detection for type II inflammatory markers in peripheral blood, further large-scale studies with prospective design are warranted for the conclusion.

## Data availability statement

The raw data supporting the conclusions of this article will be made available by the authors, without undue reservation.

## Ethics statement

The studies involving humans were approved by Ethics Committee and Institutional Review Board of The Eighth Affiliated Hospital of Sun Yat-sen University; Ethics Committee and Institutional Review Board of Dermatology Hospital of Southern Medical University. The studies were conducted in accordance with the local legislation and institutional requirements. The participants provided their written informed consent to participate in this study.

## Author contributions

BL: conception and design of study, acquisition of data, data analysis, drafting of manuscript. YW: acquisition of data, data analysis, drafting of manuscript. XW: acquisition of data, data analysis. XZ: acquisition of data. RX: conception and design of study, drafting of manuscript and critical revision. ZZ: conception and design of study, drafting of manuscript and critical revision. All authors contributed to the article and approved the submitted version.
